# The Effect of Polymer Microstructure on Encapsulation Efficiency and Release Kinetics of Citropin 1.1 from the Poly(ε-caprolactone) Microparticles

**DOI:** 10.3390/nano8070482

**Published:** 2018-06-30

**Authors:** Urszula Piotrowska, Ewa Oledzka, Wojciech Kamysz, Sławomir Białek, Marcin Sobczak

**Affiliations:** 1 Department of Biomaterials Chemistry, Chair of Analytical Chemistry and Biomaterials, Faculty of Pharmacy with the Laboratory Medicine Division, Medical University of Warsaw, Banacha 1 St., 02-097 Warsaw, Poland; eoledzka@wum.edu.pl (E.O); marcin.sobczak@wp.pl (M.S.); 2 Department of Organic Chemistry and Biochemistry, Faculty of Materials Science and Design, Kazimierz Pulaski University of Technology and Humanities in Radom, 27 Chrobrego St., 26-600 Radom, Poland; 3 Department of Inorganic Chemistry, Faculty of Pharmacy with the Laboratory Medicine Division, Medical University of Gdansk, Al. Gen. J. Hallera 107 St., 80-416 Gdansk, Poland; wojciech.kamysz@gumed.edu.pl; 4 Department of Biochemistry and Clinical Chemistry, Faculty of Pharmacy with the Laboratory Medicine Division, Medical University of Warsaw, Banacha 1 St., 02-097 Warsaw, Poland; slawomir.bialek@wum.edu.pl

**Keywords:** antimicrobial peptides, drug release, hemotoxicity, microparticles, poly(ε-caprolactone)

## Abstract

Cationic antimicrobial peptides represent a promising therapeutic option against multidrug-resistant bacteria for the treatment of local infections. However, due to their low stability and potential toxicity, there are limited possibilities for their application in clinical practice. In this study, different poly(ε-caprolactone) (PCL) microparticles (MPs) loaded with citropin 1.1 (CIT) were investigated in order to demonstrate the effect of the polymer microstructure on the encapsulation efficiency (*EE*) and kinetics of the peptide release from the newly developed devices. The characteristics of the new systems in terms of surface morphology, particle size, *EE* and zeta potential analysis, as well as the haemolytic activities of the peptide were investigated. The *in vitro* release kinetics of CIT from the MPs was also investigated. CIT loading was favoured by a high content of negative charged linear polymer chains in the PCL structure. The presence of non-charged, amorphous macrocycle domains results in faster degradation of the PCL matrix. Depending on the crystallinity of the PCL, the peptide release exhibited a near-zero-order or near-first-order profile with no “burst release”. The results indicated that CIT-loaded PCL MPs could potentially be a promising drug delivery system (DDS) for the treatment of local infections.

## 1. Introduction

Cationic antimicrobial peptides (AMPs) represent a promising group of active pharmaceutical ingredients (APIs) for a new therapeutic option against multidrug-resistant bacteria in the treatment of local infections. Furthermore, depending on their structure and origin, AMPs may also show antitumor, angiogenic, immunomodulatory and wound healing activities [1,2].

Despite their promising therapeutic potential, as low molecular weight cationic molecules, peptides have relatively low stability *in vivo* due to a more favourable interaction with the anionic species of body fluids [3]. As a consequence, they are rapidly removed from bloodstream circulation, resulting in a decrease in their therapeutic concentrations. Moreover, a high content of hydrophobic amino acids in their structure remains a critical factor for determining their potential toxicity [4].

To overcome the above shortcomings, biodegradable polymeric carriers nano- (NPs) and microparticles (MPs) can be designed for controlled peptide release, which is very important in the case of biomedical devices. Polymers could enhance the stability of the drug and decrease its toxic effects *in vivo*. Due to their non-toxicity and non-immunogenicity, the most commonly used biodegradable polymers as matrices for drug delivery are aliphatic polyesters: poly(ε-caprolactone), polylactide, polyglycolide and their copolymers [5]. The encapsulation efficiency (*EE*) and release kinetics of the AMPs from the biodegradable polymeric matrices depend on the kind of polymer, its microstructure (degree of crystallinity, *X*_c_), number averge molecular weight (*M*_n_), dyspersity index (*Đ*) and the peptide characteristics: molecular weight, net charge and hydrophilic/hydrophobic properties. Different methods have previously been reported for preparing polymeric NPs and MPs: emulsion-based methods, nanoprecipitation, salting out, spray-drying, miscellaneous methods [6], etc.

PLGA/chitosan composite [7] and PEG-PLGA [8] have recently been investigated as polymeric MPs drug delivery system (DDS) for KSL-W (KKVVFWVKFK-CONH_2_) [7] and cathelicidin-BF (KFFRKLKKSVKKRAKEFFKKPRVIGVSIPF) [8]. However, in the case of cathelicidin-BF, three release phases with an initial burst-release phase of peptide have been observed [8]. 

This study is a continuation of our previous research [9] on the synthesis and investigation of the structural, physicochemical and biological properties of the PCLs matrices for prolonged peptide release. In this paper, medium-term delivery systems of citropin 1.1 (CIT, amino acids sequence: GLFDVIKKVASVIGGL) were prepared from poly(ε-caprolactone) (PCL) matrices using the solvent evaporation method. The main goal of our current paper is to evaluate the crucial parameters responsible for the *EE* and release kinetics of cationic peptide from the PCLs MPs with various microstructures.

## 2. Materials and Methods 

### 2.1. Materials

PCL-1 (*M*_n_ = 6100 g·mol^−1^; *Đ* = 1.8; *X*_c_ = 50.5%) and PCL-2 (*M*_n_ = 5700 g·mol^−1^, *Đ* = 1.5, *X*_c_ = 56.3%) matrices were obtained by the enzymatic ring-opening polymerization (eROP) of ε-caprolactone (CL) in the presence of *Candida antarctica* lipase B (CALB) as catalyst in two ionic liquids (ILs): [bmim][NTf_2_] and [bmim][PF_6_] according to our previously described method [9]. CIT (≥ 95%) was purchased from Lipopharm.pl (Gdansk, Poland) [10]. Dichloromethane (DCM) (Avantor, Gliwice, Poland), phosphate buffered saline (PBS, Sigma, Poznan, Poland) and poly(vinyl alcohol) (PVA, *M*_w_ 13,000–23,000; 87–89% hydrolyzed) (Aldrich, Poznan, Poland) were used as received.

#### Spectroscopic Data of PCLs Matrices

^1^H-NMR (CDCl_3_, δ, ppm): 4.07 [2H, t, –CH_2_CH_2_CH_2_CH_2_C**H_2_**OC(O)–], 2.32 [2H, t, –CH_2_CH_2_CH_2_CH_2_C**H_2_**COO–], 1.67 [4H, m, –CH_2_C**H_2_**CH_2_C**H_2_**CH_2_COO–], 1.39 [2H, m, –CH_2_CH_2_C**H_2_**CH_2_CH_2_COO–]; ^13^C–NMR (CDCl_3_, δ, ppm): 173.7 [–**C**(O)O–], 64.3 [–CH_2_CH_2_CH_2_CH_2_**C**H_2_OC(O)–], 34.3 [–CH_2_CH_2_CH_2_CH_2_**C**H_2_COO–], 28.5 [–CH_2_CH_2_CH_2_**C**H_2_CH_2_OC(O)–], 25.7 [–CH_2_CH_2_CH_2_**C**H_2_CH_2_COO–], 24.7 [–CH_2_CH_2_**C**H_2_CH_2_CH_2_COO–];

FT-IR (KBr, cm^−1^): 2938 (ν_as_CH_2_), 2866 (ν_as_CH_3_), 1727 (νC=O), 1245 (νC–O)

### 2.2. Single Emulsion Microspheres’ Preparation

PCL (50 mg) was dissolved in 2.5 mL of DCM and added dropwise to an aqueous solution of 5% (*w/v*) PVA. The dispersion was homogenized (SilentCrusher, Heidolph Instruments GmbH & Co, Schwabach, Germany) for 1 min at 10,000 rpm to reduce droplet size and then mixed for 4 h at 400 rpm in order to harden the microspheres. The mixture was then washed with distilled water and centrifuged at 3,500 rpm for 30 min (MPW-54 centrifuge, MPW MED. INSTRUMENTS, Warsaw, Poland) to thoroughly remove the PVA residue. Then, the MPs were preserved at 4 °C.

### 2.3. Double Emulsion Microspheres’ Preparation 

The CIT (1 mg) was dissolved in PBS (1 mL) and the solution was added to the 5 mL (1.5% *w/v* or 3.0% *w/v*) of the PCL/DCM solution to make a primary (W/O) emulsion using a homogenizer (SilentCrusher, Heidolph Instruments GmbH & Co, Schwabach, Germany) for 1 min at 10,000 rpm. The primary (W/O) emulsion was added dropwise to the 8 mL of 5% (*w/v*) PVA/distilled water solution and homogenized for 5 min at 10,000 rpm to produce a final double (W/O/W) emulsion. The 60 mL of 1% (*w/v*) PVA/distilled water solution was then added dropwise and stirred mechanically for 4 h at 400 rpm at room temperature until the organic solvent was thoroughly evaporated. Finally, the mixture was washed further with distilled water under centrifugation to thoroughly remove PVA. Then, the MPs were preserved at 4 °C.

### 2.4. Determination of the Encapsulation Efficiency

The microspheres were dissolved in DCM and the resulting solution was added to 1 mL of PBS and stirred for 24 h. The mixture was then centrifuged for 10 min at 35,000 rpm and the supernatant aqueous phase containing CIT was then quantified by the high-performance liquid chromatography (HPLC) method, according to previously described procedures [10]. The samples were prepared in triplicate. The peptide *EE* was calculated using the following equation (Equation (1)):

*EE* = (measured drug content/theoretical drug content) × 100 (1)

### 2.5. In Vitro Studies of Citropin 1.1 Release From the Microspheres

The CIT-loaded PCL microspheres were incubated with 1mL of PBS (pH 7.4) at 37 °C. The samples were centrifuged at 3,500 rpm for 10 min and a supernatant from each run was removed for analysis at the selected time intervals, after which this was replaced with a new PBS solution. The quantity of CIT released from the PCL microspheres was analysed using the HPLC method. The samples were prepared in triplicate.

Release data were analyzed on the basis of zero-order and first-order mathematical models as well as the Higuchi model and the Korsmeyer–Peppas model (Table 1) [11,12,13].

### 2.6. Hemolytic Activity of the Peptide and Calculation of the Therapeutic Index

The haemolytic activity of CIT was measured as the amount of haemoglobin released according to Reference [14] with a small own modification. Human Red Blood Cells (hRBC) with EDTA was rinsed three times with saline (0.9% NaCl) using centrifugation for 10 min at 800 x g and was then re-suspended in saline. Serial dilution of the peptides (64, 128, 192, 256 and 512 μg·mL^−1^) was performed in saline on 96-well plates. A stock solution of hRBC was then added to obtain a final volume of 100 μL with a 4% concentration of erythrocytes (*v/v*). The control wells for haemolysis of 0 and 100% contained hRBC suspended in saline and 1% Triton-X 100, respectively. The plates were then incubated for 60 min at 37 °C and then centrifuged at 800 x g for 10 min at 4 °C. Following centrifugation, the supernatant was carefully suspended in new microplates and the release of haemoglobin was monitored and the absorbance was measured at 540 nm (SYNERGY Mx Microplate Spectrophotometer, BioTek, Winooski, VT, USA). All experiments were carried out in triplicate.

The therapeutic Index *(TI*) of the CIT in our investigations was calculated using the following equation (Equation (2)):

*TI* = *MHC*/*MIC* (2)

where *MHC*: minimal hemolytic concentration of CIT to hRBC; *MIC*: minimal inhibitory concentration of CIT to methilicin-resistant *S. aureus* (MRSA) strains, commonly known as the major pathogen in surgical wound infections.

### 2.7. Measurements

The polymerization products were characterized by means of ^1^H and ^13^C NMR (Varian 300 MHz) at room temperature, with CDCl_3_ as solvent. The infrared Fourier transform (FT-IR) spectra were measured from KBr pellets (Perkin Elmer spectrometer, Perkin Elmer, Warsaw, Poland). 

HPLC measurements were performed using WATERS apparatus (Milford, MA, USA) with ZORBAX column (1.8 μm, SB-C18, 4.6 mm × 50 mm, Agilent, Santa Clara, CA, USA). The mobile phase consisted of water (0.1% formic acid) and acetonitryl (0.1% formic acid) at the flow 1 mL·min^−1^ [10].

The morphological assessment of the MPs was investigated by scanning electron microscope (SEM) FEI Quanta 250 FEG (FEI Inc., Eindhoven, The Netherlands). 

The average particle size and zeta potential of the microspheres were determined by a dynamic light scattering (DLS) technique using a Zetasizer Nano ZS instrument (Malvern Instruments, Westborough, MA, USA) equipped with red laser at a wavelength of 633 nm and scattering angle of 173° at 25 °C.

## 3. Results and Discussion

### 3.1. Morphology, Size and Zeta Potential of MPs

**Figure 1:**
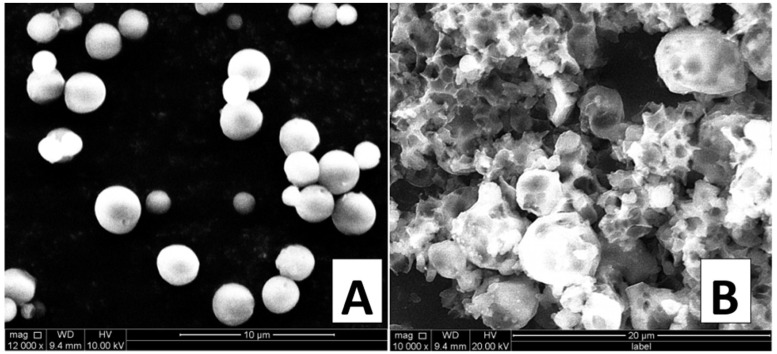
SEM micrographs of CIT-loaded PCLs MPs obtained using a double emulsion, at different amounts of the aqueous external phase: (**A**) optimum amount of the aqueous external phase in secondary emulsion; (**B**) smaller amount of the aqueous external phase in secondary emulsion.

The results of the SEM investigations revealed that spherical, non-porous and smooth PCL MPs were successfully obtained. There were no morphological differences between AMPs-loaded and placebo MPs prepared using both the single- and double-emulsion methods. The crucial parameter for the preparation of non-porous MPs was the appropriate amount of the aqueous phase in the final emulsion. Moreover, moderate evaporation of DCM during the last stage of the process is also required to obtain smooth PCL MPs [15]. Figure 1A presents the SEM micrograph of the CIT-loaded PCL MPs obtained using the optimum amount of the aqueous external phase to diffuse DCM in the emulsion. Figure 1B shows the SEM micrograph of the MPs prepared using the same procedure as reported in the ‘Experimental’ section but with a smaller amount of the aqueous external phase. Consequently, the fast evaporation of an organic solvent causes leads to irregular, high porous and rough PCL MPs being obtained.

The size and surface charge of the polymeric MPs are the key factors responsible for their cellular uptake and toxicity effect *in vivo*. The mean diameter of the MPs prepared using a single emulsion method ranged from 3.69 to 3.92 µm with a narrow particle size distribution (0.22–0.24) in the case of both matrices (Table 2). The mean diameter of the placebo and the CIT-loaded MPs, prepared using a double-emulsion solvent evaporation method, was slightly higher and ranged from 3.90 to 5.56 µm with a dyspersity values of 0.13–0.38. 

MPs are able to prevent the toxic effects associated with the electrostatic interaction between negative-charged glycoproteins molecules embedded in the hRBC membrane and cationic APIs [16]. Moreover, surface charge affects the stability of the MPs in suspensions. Zeta potential measurements revealed that all MPs have negative surface charge (from −19.7 to −12.2 mV), which could be explained by the presence of the carboxylic end-group of the linear PCLs chains (Table 2) [17].

### 3.2. Encapsulation Efficiency of CIT

The *EE* of the peptide is a function of the characteristics of the peptide and the MPs microstructure. This depends on the electrostatic and hydrophobic interactions between polymer and cationic peptides. In our experiments, the preparation of CIT-loaded MPs was successfully achieved using a double-emulsion method, which is widely used for the encapsulation of water-soluble APIs [18,19,20].

As shown in Table 2, the *EE* of CIT was in the range of 42–52%. MPs were obtained from two PCLs matrices with varying crystallinity. MP-PCL-1-CIT-0.5 (0.5% initial contain of CIT in PCL) and MP-PCL-1-CIT-1.0 (1.0% initial contain of CIT in PCL) were obtained from PCL-1 (*M_n_* = 6100 g·mol^−1^, *X_c_* = 50.5%). In turn, MP-PCL-2-CIT-0.5 (0.5% initial contain of CIT in PCL) and MP-PCL-2-CIT-1.0 (1.0% initial contain of CIT in PCL) were obtained from PCL-2 (*M_n_* = 5700 g·mol^−1^, *X_c_* = 56.3%). The decrease in CIT-loading between MP-PCL-1-CIT-0.5 and MP-PCL-1-CIT-1.0 and between MP-PCL-2-CIT-0.5 and MP-PCL-2-CIT-1.0 is a logical consequence of increasing the initial amount of PCL from 100 to 200 mg during the preparation of the primary (W/O) emulsion. 

Moreover, we noted some differences in the *EE* of CIT between MPs originating from polymers with different microstructures. In the case of PCL-1, the *EE* was in the range of 42–47% and was lower than that of PCL-2 (51–52%). This is probably due to the differences in the polymer microstructure, which consists of non-charged macrocycles and negatively charged linear PCL chains. The presence of the negatively charged molecules in the MPs structure allows for an electrostatic interaction between the carboxyl end-group of the linear polymer and the cationic peptides, which results in enhanced *EE*. PCL-1 with *X_c_* of 50.5% that have a high content of non-charged macrocycles in their structures (ca. 35% [9]), thus the *EE* in this case was lower. In contrast, we noted higher *EE* values for MP-PCL-2 (*X_c_* = 56.3%) with a lower content of macrocyclic chains (19% [9]).

### 3.3. The In Vitro Kinetics Release of CIP from PCL MPs

**Figure 2:**
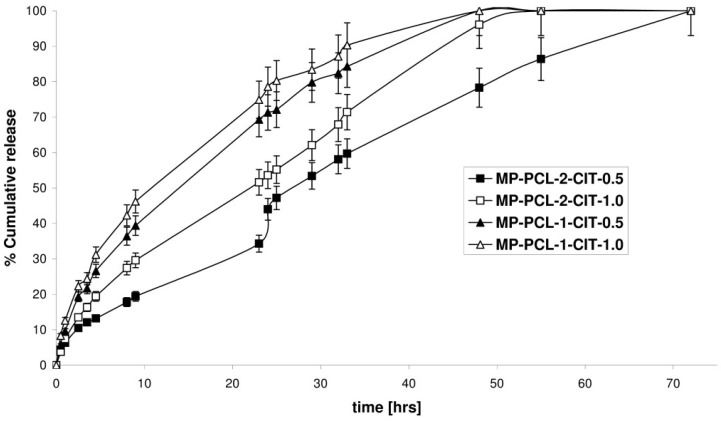
Cumulative release of CIT from the MP-PCL-1-CIT-0.5, MP-PCL-1-CIT-1.0, MP-PCL-2-CIT-0.5 and MP-PCL-2-CIT-1.0 during 72 h (each point represents the mean ± SD of three points).

The in vitro kinetic release of CIT from the obtained MPs was determined at pH 7.4, 37 °C over about 72 h. The in vitro release peptide profiles are presented in Figure 2. The ordinate of the plot was calculated based on the cumulative amount of CIT released. MPs were obtained from two PCL matrices with varying crystallinity. Our main intention was to examine how the kinetics of CIT release depends on the crystallinity of the polymeric matrices. It has, in fact, been reported that PCL degradation is very slow in an aqueous medium because of its semi-crystallinity and hydrophobicity. However, water can penetrate easily into the amorphous regions of the polymer matrix, facilitating the release of the drug by diffusion [21,22,23]. A comparison of CIT release from MPs with varying amounts of the peptide was also analyzed.

It was found that the difference in the observed release rate for the MPs was mainly attributed to the difference in the *X_c_* of the PCL matrices. The rate of in vitro peptide release increased as the *X_c_* of the matrices decreased. The percentage of the peptide released after 33 h of incubation was about 59.7% for the MP-PCL-2-CIT-0.5, 71.4% for the MP-PCL-2-CIT-1.0, 84.3% for theMP-PCL-1-CIT-0.5 and 90.3% for the MP-PCL-1-CIT-1.0. It was also observed that the release rates varied depending on the amount of CIT encapsulated in the MPs. The release was slower for MPs with a higher amount of peptide, which could be attributable to the high hydrophobicity nature of the CIT (ca. 56% of hydrophobic amino acids in sequence). MPs consisting of different initial amounts of the peptide released 100% of CIT in about 72, 55, 48 and 33–48 h for MP-PCL-2-CIT-0.5 (51.5% of the initial amount of peptide), MP-PCL-2-CIT-1.0 (50.5% of the initial amount of peptide), MP-PCL-1-CIT-0.5 (46.8% of the initial amount of peptide) and MP-PCL-1-CIT-1.0 (42.2% of the initial amount of peptide).

The release data points were subjected to zero-order and first-order mathematical models, as well as the Higuchi model and the Korsmeyer–Peppas model, to evaluate the kinetics and release mechanisms of the CIT from the obtained MPs (Table 3). 

In the Korsmeyer–Peppas model, the value of *n* characterizes the release mechanism of the peptide. According to the Korsmeyer–Peppas model, in the case of spheres, 0.43 ≤ *n* corresponds to a Fickian diffusion mechanism, 0.43 < *n* < 0.85 to non-Fickian transport, *n* = 0.85 to case II transport and *n* > 0.85 to super case II transport [11,12,13,14]. It was shown that the CIT release from MP-PCL-2-CIT-1.0 occurred in one-step. The peptide was released with a near-zero-order kinetics (*R*^2^ = 0.9878). This suggests that CIT release from the MP-PCL-2-CIT-1.0 is a highly controlled process. Interestingly, the peptide was released in two steps from MP-PCL-2-CIT-0.5. However, CIT was also released in accordance to the near-zero-order kinetics form MPs in both phases. The controlled peptide release profiles were obtained with no significant “burst release”. High *R*^2^ values (0.9708 and 0.9835) were obtained for the near-zero-order kinetics model. The MPs obtained from the PCL-1 show a slightly less controlled release profile. The CIT release exhibited a more near-first-order release profile. The *R*^2^ values were 0.9854 and 0.9814 for MP-PCL-1-CIT-0.5 and MP-PCL-1-CIT-1.0, respectively. The *R*^2^ values of the Korsmeyer-Peppas model were high (from 0.9818 to 0.9893) for all MPs. The *n* value was above 0.85 for all MP-PCL-2-CIT-0.5 (in phase II), MP-PCL-2-CIT-1.0, MP-PCL-1-CIT-0.5 and MP-PCL-1-CIT-1.0. This suggests that the CIT release was governed mainly by super case II transport. However, in the case of phase II of MP-PCL-2-CIT-0.5 (in phase I), the *n* value was 0.79, which indicates that the anomalous transport (non-Fickian) dominates in these systems.

As presented in Table 3, the data was also suited to the Higuchi model, with correlation coefficients of 0.9714–0.9887. According to this model, the liquid penetrates the MPs and dissolves the embedded CIT, thus the peptide release seems to be a process predominately controlled by diffusion. As commonly known, drug release from nano- or microparticles generally follows diffusion/degradation or a combination of diffusion and degradation-mediated release phenomena. It was shown that CIT was released from MP-PCL-2-CIT-0.5 and MP-PCL-2-CIT-1.0 according to near-zero-order kinetics in case II transport or super case II transport. In turn, in the cases of MP-PCL-1-CIT-0.5 and MP-PCL-1-CIT-1.0, the peptide was released according to near-first-order kinetics by super case II transport. Analysis of the models suggested that peptide release depended more on the erosion of the PCL than on the diffusion process. However, as is known, mathematical models do not provide an exact insight into the mechanism of peptide release.

### 3.4. Hemolytic Activity of Peptides and Calculation of the Therapeutic Index

**Figure 3:**
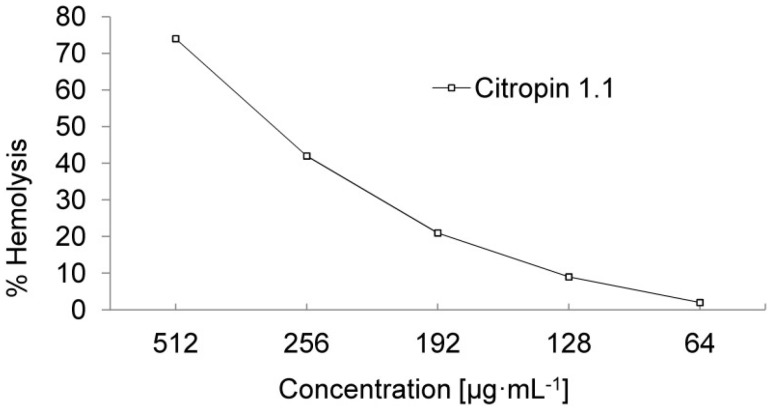
Hemolysis of hRBC as a function of CIT concentration.

Membrane-active peptides with a high content of hydrophobic amino acids in their sequences could interact with eukaryotic cells and provide some toxic effects in vivo. The mechanisms of their interaction depend on the type of the target cells. In the case of hRBC, the toxic effect is a result of the pore formation of the peptide in cell membranes, whereas AMPs induce apoptosis in Human White Blood Cells (hWBC) [24]. In our study, we chose hRBC as the test cells to determine the *TI* of CIT according to Chen et al. [25] *TI* is a widely used parameter for determining the AMPs specificity towards prokaryotic cells [24]. *TI* is described as the ratio of minimal haemolytic concentration (*MHC*) and minimal inhibitory concentration (*MIC*), thus larger *TI* values indicate the greater specificity of the peptides toward the bacteria being tested. In our investigation, we considered the *TI* in relation to the MIC of the methicillin-resistant *S. aureus* (MRSA) strains. *S. aureus* is commonly known to be the major pathogen in surgical wound infections. The *MIC* values of CIT toward MRSA were in the range of 1–16 µg·mL^−1^, as previously reported [26]. The results of toxicological assays (Figure 3) revealed 9% haemolysis at 128 μg·mL^−1^ and 74% haemolysis at 512 μg·mL^−1^. 

To calculate *TI* we chose the highest reported *MIC* value (16 μg·mL^−1^), and the *TI* value of the CIT in relation to MRSA strains was eight. The results revealed that CIT indicates a relative high specify toward MRSA, hence it could be used as a potential therapeutic agent against multidrug-resistant bacteria in surgical infection.

**Figure 4:**
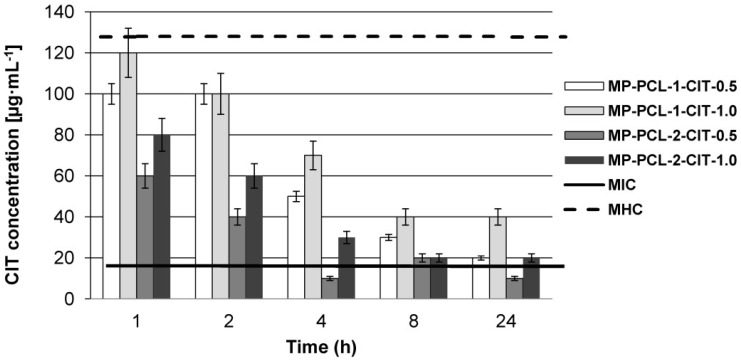
Peptide concentrations at selected hourly intervals during first 24 h of incubation in PBS solution at 37 °C and pH 7.4.

Figure 4 shows peptide concentrations at selected hourly intervals during the first 24 h of incubation in a PBS solution at 37 °C and pH 7.4. From a therapeutic point of view, the best matrices for CIT release were MP-PCL-1-CIT-0.5 and MP-PCL-2-CIT-1.0. In these cases, CIT reached the therapeutic concentration within the first hour of release. This confirmed our earlier assumption that CIT release from the selected MPs is a highly controlled process. In the case of MP-PCL-2-CIT-1.0 CIT release was very low ca. 10 µg·mL^−1^ (MIC_MRSA_ = 16 µg·mL^−1^) at four and 24 h. Moreover, for MP-PCL-1-CIT-1.0 devices, CIT release was high and exceeded the MHC value (MHC = 128 µg·mL^−1^) during the first hour. None of the tested matrices release CIT at concentration above HC_50_ (HC_50_ = 256 µg·mL^−1^). 

## 4. Conclusions

Poly(ε-caprolactone) (PCL) based micropartices (MPs) loaded with citropin 1.1 (CIT) were successfully prepared and investigated. MPs were obtained from PCL with varying crystallinity and different contents of CIT. It was found that CIT was released from the obtained MPs with rather controlled kinetics. In some cases, the peptide was released from the carriers with a near-zero-order release kinetics. It is also worth noting that peptide “burst release” was not observed. Importantly, the MPs loaded with CIT exhibited antimicrobial activity during *c.a.* 72 h of the degradation process. The development of the obtained MPS should be of great interest in the delivery systems of antimicrobial agents.

## Figures and Tables

**Table 1:** Mathematical models of kinetic or release mechanism.

Mathematical Model	Equation
Zero-order model	*F* = *kt*
First-order model	log *F* = log *F*_0_ – *kt*·(2.303)^−1^
Higuchi model	*F*= *kt*^1/2^
Korsmeyer-Peppas model	*F*=*kt^n^ (F < 0.6)*

*F*: fraction of drug released up to time (*t*), *F_0_*: initial concentration of drug, *k*: constant of the mathematical models, *n*—exponent of Korsmeyer–Peppas model.

**Table 2:** Characterization of PCLs MPs.

Entry	Method ^1^	Initial Contain of CIT in PCL (%, *w/w*)	Particle Size (µm)	*Đ* ^2^	‎Zeta Potential (mV)	*EE*^3^ (%)
MP-PCL-1-1	A	0	3.69 ± 1.88	0.22	−19.3 ± 5.3	-
MP-PCL-1-2	B	0	5.50 ± 1.70	0.25	−13.1 ± 3.9	-
MP-PCL-1-CIT-0.5	B	0.50	5.46 ± 0.71	0.38	−16.0 ± 4.3	46.8 ± 1.8
MP-PCL-1-3	B	0	3.90 ± 1.81	0.18	−17.5 ± 4.9	-
MP-PCL-1-CIT-1.0	B	1.02	4.67 ± 2.10	0.23	−16.8 ± 5.2	42.2 ± 1.2
MP-PCL-2-1	A	0	3.92 ± 2.13	0.24	−15.6 ± 5.7	-
MP-PCL-2-2	B	0	4.67 ± 2.00	0.21	−12.2 ± 3.5	-
MP-PCL-2-CIT-0.5	B	0.51	5.06 ± 1.77	0.13	−17.9 ± 5.7	51.5 ± 1.4
MP-PCL-2-3	B	0	4.52 ± 0.89	0.13	−19.7 ± 5.4	-
MP-PCL-2-CIT-1.0	B	1.04	5.56 ± 1.61	0.12	−19.5 ± 6.4	50.5 ± 1.7

^1^ single (A) and double (B) emulsion solvent-evaporation method to obtain PCL MPs; ^2^ dyspersity index; ^3^ encapsulation efficiency.

**Table 3:** Analysis data of CIT release from MPs.

No.	Zero Order Model	First Order Model	Higuchi Model	Korsmeyer-Peppas Model	
	*R* ^2^	*R* ^2^	*R* ^2^	*R* ^2^	*n*
MP-PCL-2-CIT-0.5	-	-	-	-	-
Phase I	0.9708	0.9670	0.9822	0.9848	0.79
Phase II	0.9835	0.9689	0.9866	0.9818	> 0.85
MP-PCL-2-CIT-1.0	0.9878	0.8173	0.9714	0.9893	> 0.85
MP-PCL-1-CIT-0.5	0.9562	0.9854	0.9887	0.9855	> 0.85
MP-PCL-1-CIT-1.0	0.9289	0.9814	0.9831	0.9836	> 0.85
